# Supplementary material for: Constructing a Flood-Adaptive Ecological Security Pattern from the Perspective of Ecological Resilience: A Case Study of the Main Urban Area in Wuhan
Source: Int J Environ Res Public Health. 2022 Dec 26;20(1):385. doi: 10.3390/ijerph20010385 (PMC9820015; doi:10.3390/ijerph20010385)
Supplement: Supplementary file 1 [file ijerph-20-00385-s001.zip › Table S1 Specific location of the corridor.pdf]

There are 9 ecological corridors found in the Hankou Ecological Zone：

| corridor | Location |
|---|---|
| ① | Tazi Lake–Tazihu East Road–Harmony Avenue–Changgang Road–Jiangfa Road–Garden Expo Park–Fengmei Road–Chang'an Road–Hubei University of Police–Gutian 3rd Road–Hanjiang River–Yangcheng–Hanjiangyuan–Huayuanwan Road–Fairy Mountain Road–Longyang Lake |
| ② | Tazi Lake–Tazihu East Road–Zhongyi Road–Huangxiaohe Road–Jianshe Avenue–Chestnut Lake–Huanzi Lake Group |
| ③ | Tazihu–Tazihu East Road–Zhongyi Road–Huangpu Street–Yangtze River |
| ④ | Chestnut lake–Huanzi Lake Group–Jianshe Avenue–Mengze Lake Park |
| ⑤ | Small South Lake Park–Xinhua Road–Changjiang Daily Road–Jiefang Avenue–Macau Road–Sanyang Road–Yangtze River |
| ⑥ | Mengze Lake Park–The above-ground part of Metro Line 3–Jiefang Avenue–Second Ring Line |
| ⑦ | Small South Lake Park–Xinhua Road–Shuangdong Main Street–Jinghan Avenue–Wusheng Road Overpass–Parrot Avenue–Moon Lake |
| ⑧ | Small South Lake Park–Xinhua Road–Shuangdong Main Street–Friendship Road–Friendship South Road–Hanjiang River |
| ⑨ | Jiefang Avenue–Hanxi Road–Hanjiang River–Qintai Avenue–Moon Lake Bridge–Moon Lake |

16 ecological corridors were found in the Hanyang Ecological Zone:

| corridor | Location |
|---|---|
| ⑩ | Longyang Lake–Moshui Lake North Road–Yulong Road–Hanyang Avenue–Jinlong Road–Rose Street–Longyang Avenue |
| ⑪ | Wuqin Road–Hanyang Avenue–Ma Canghu Road–Moshui Lake |
| ⑫ | Longyang Lake–Majialing Road–Moshui Lake |
| ⑬ | Moshui Lake–Sixin Middle Road–Yangsigang Expressway–Sixin North Road–Yangtze River |
| ⑭ | Longyang Lake–Triangle Lake |
| ⑮ | Moshui Lake–Sixin Middle Road–Wandao Wetland Park–Sixin Avenue–Fangcao Road–North Taizi Lake |
| ⑯ | Triangle Lake–North Taizi Lake |
| ⑰ | Triangle Lake–Boxue Road–Bihu Road–Houguan Lake |
| ⑱ | Triangle Lake–Xinmin River–South Taizi Lake |
| ⑲ | North Taizi Lake–South Third Ring Wetland Park–Haitang Road–Taizi Lake North Road–Jiangcheng Avenue–South Taizi Lake |
| ⑳ | Moshui Lake–Jiangxinyuan Road–Guobo Avenue–Second Ring Road–Yangtze River |
| ㉑ | South Taizi Lake–Dunkou Road–Guobo Avenue–Sixin North Road–Yangtze River |
| ㉒ | The north part of Houguan Lake–Guanhu Road–The south part of Houguan Lake |
| ㉓ | The south part of Houguan Lake–Lianhu Road–Dongfeng Avenue–South |

| | |
|---|---|
| | Checheng Road–Tanghu |
| ㉔ | Tanghu–Checheng Avenue–Wanjia Lake |
| ㉕ | Wanjia Lake–Wanjiahu Middle Road–Binhu West Road–South Taizi Lake |

7 ecological corridors were found in the Wuchang Ecological Zone:

| corridor | Location |
|---|---|
| ㉖ | East Lake–Chu River–Shahu–Chuhan Road–Yangtze River |
| ㉗ | South Lake–Wuhan Institute of Technology (Yongjun Road)–East Lake–Yangchun Lake Road–Yangchun Lake–East Lake Port–Qingshan Park–Liulin Park |
| ㉘ | Yangtze River–Zhonghua Road–Shouyi Park–Wuluo Road–Jiedaokou |
| ㉙ | 3rd Ring Road–Guangxia West Road–Guangxia Road–Fengsheng Road–Fengsheng East Road–Maotan Park–Living Water Park–River Tour Company–Zhongshan Road–Gongzheng Road–Shahu |
| ㉚ | East Lake–Central China Normal University–Guiyuan Road–Wuhan University of Technology–Jiedaokou–Luoshi Road–East Lake; ㉛ Yanxi Lake–Yanxi Lake Tunnel–East Lake |
| ㉛ | Yanxi Lake–Yanxi Lake Tunnel–East Lake |
| ㉜ | Yezhi Lake–Nanhu Avenue–Hubei University of Technology–Wuchang Institute of Technology–Metro Line 2 (under construction)–Yangtze River |
