# Supplementary figures and images for: Constructing a Flood-Adaptive Ecological Security Pattern from the Perspective of Ecological Resilience: A Case Study of the Main Urban Area in Wuhan
Source: Int J Environ Res Public Health. 2022 Dec 26;20(1):385. doi: 10.3390/ijerph20010385 (PMC9820015; doi:10.3390/ijerph20010385)

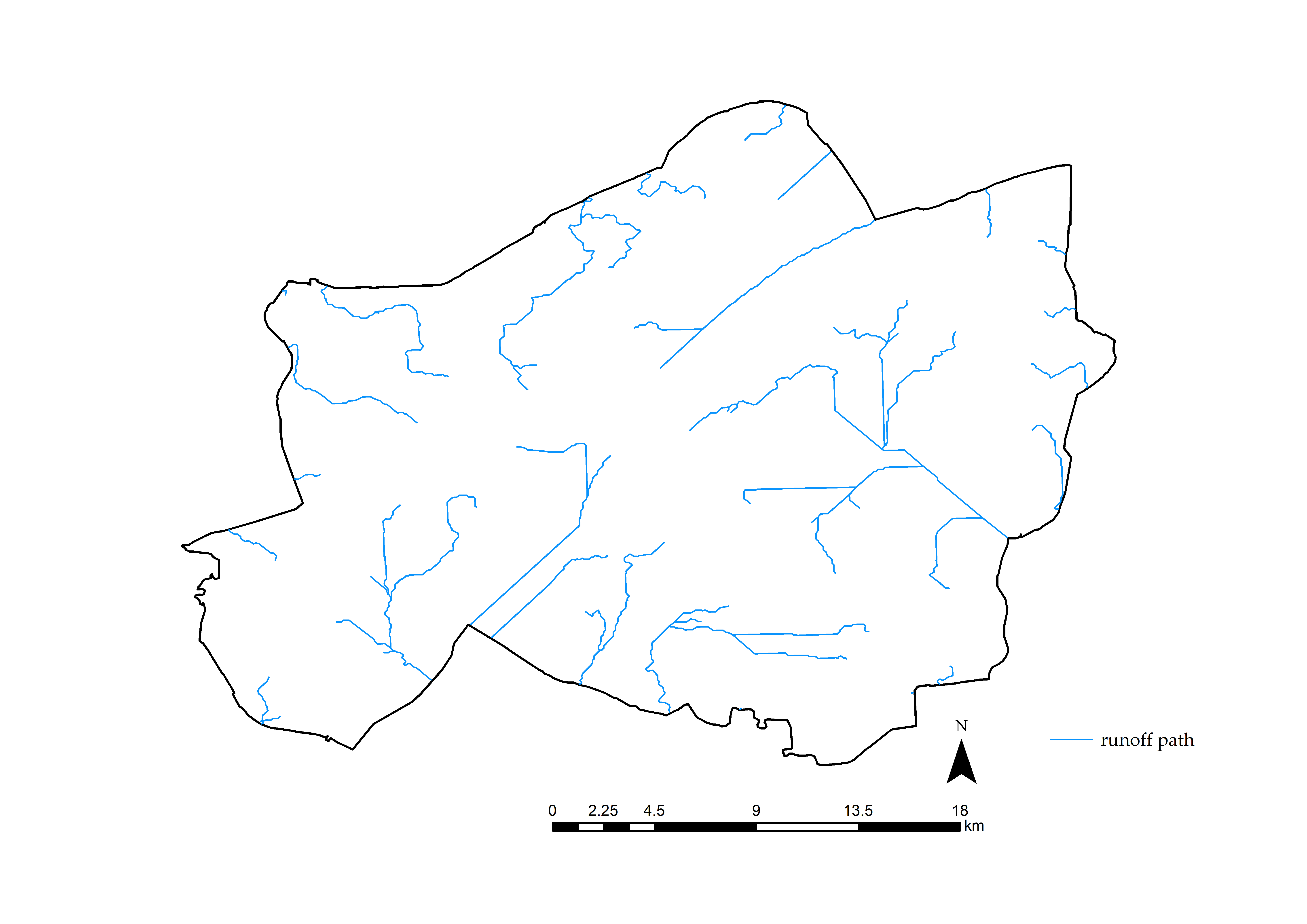

Supplement: Supplementary file 1 [file ijerph-20-00385-s001.zip › Figure S1 runoff path distribution.png]

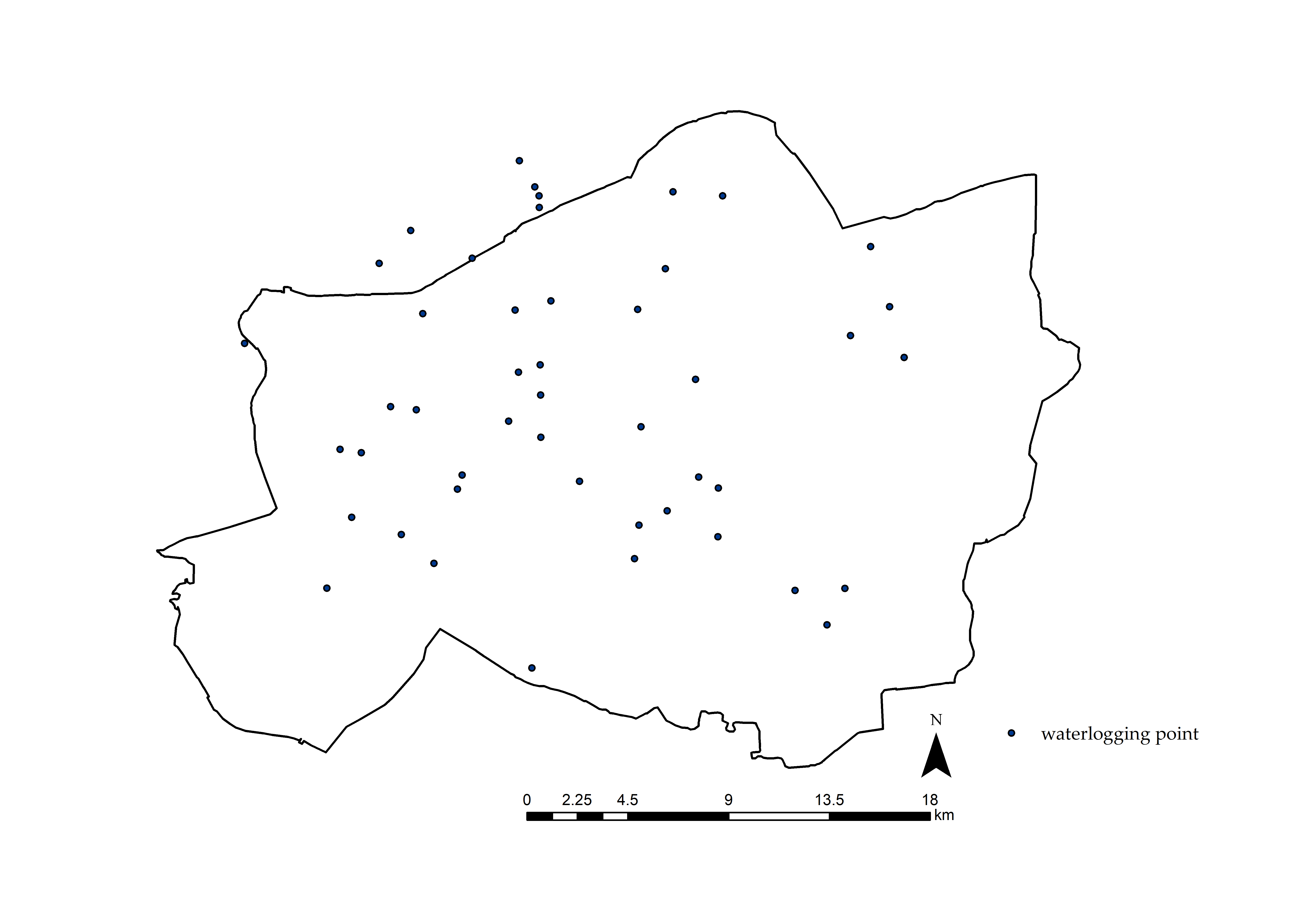

Supplement: Supplementary file 1 [file ijerph-20-00385-s001.zip › Figure S2 Waterlogging risk points distribution.png]
